# Printed Graphene Derivative Circuits as Passive Electrical Filters

**DOI:** 10.3390/nano8020123

**Published:** 2018-02-23

**Authors:** Dogan Sinar, George K. Knopf

**Affiliations:** Department of Mechanical and Materials Engineering, The University of Western Ontario, London, ON N6A 5B9, Canada; dsinar@uwo.ca

**Keywords:** inkjet printing, graphene, carboxymethyl cellulose, *RC* low pass filters, flexible electronics

## Abstract

The objective of this study is to inkjet print resistor-capacitor (*RC*) low pass electrical filters, using a novel water-based cellulose graphene ink, and compare the voltage-frequency and transient behavior to equivalent circuits constructed from discrete passive components. The synthesized non-toxic graphene-carboxymethyl cellulose (G-CMC) ink is deposited on mechanically flexible polyimide substrates using a customized printer that dispenses functionalized aqueous solutions. The design of the printed first-order and second-order low-pass *RC* filters incorporate resistive traces and interdigitated capacitors. Low pass filter characteristics, such as time constant, cut-off frequency and roll-off rate, are determined for comparative analysis. Experiments demonstrate that for low frequency applications (<100 kHz) the printed graphene derivative circuits performed as well as the circuits constructed from discrete resistors and capacitors for both low pass filter and *RC* integrator applications. The impact of mechanical stress due to bending on the electrical performance of the flexible printed circuits is also investigated.

## 1. Introduction

Over the past decade printable electronics have received considerable attention among researchers for the fabrication of low cost passive filters and sensors. Since very small amounts of functional material can be deposited only at the required locations on the substrate, additive fabrication processes are often used. In particular, inkjet-printed functional materials can be self-assembled on non-absorbent substrates without the need of extreme temperature annealing or harsh chemical etching processes. The direct deposition process also allows various rigid (e.g., glass) and non-rigid (e.g., polymers, paper) substrates to be used. Furthermore, inkjet printing systems are computer-numerically controlled such that thin film circuits are fabricated without direct mechanical contact between the deposition tool and substrate. The use of computerized systems to design and fabricate printed devices also reduces human error and enables circuit or sensor customization. Compared to other alternatives such as photolithography, inkjet printing requires lower infrastructure costs and produces far less hazardous waste materials. In addition, prototyping and implementing design revisions can be made quickly without requiring the construction of new photolithography or stencil masks. This requirement is especially useful for creating mechanically flexible electronics for communication and signal processing circuits in customized wearable devices where a high degree of variation may exist in the circuit parameters. For example, radio frequency identification (RFID) antennas and passive electrical filters are an integral part of many sensors and signal processing systems. Small variations in circuit geometry define resonance frequency of RFID antennas while resistive and capacitive component parameters define cutoff frequency (*f_c_*) of passive electrical filters.

Although inkjet printing can simplify design and fabrication, the performance of printed circuits ultimately depends on the availability of functional materials. Inkjet-printed thin film electronics are largely based on metal inks [1,2,3] deposited on rigid substrates such as glass or silica. However, alternative functional inks based on polymers [4,5], carbon nanotubes (CNTs) [6], and graphene (G)/graphene oxide (GO) [7,8] have been demonstrated over the past decade. For example, Chen et al. presented a study based on inkjet printing of conductive poly(3,4-ethylenedioxythiophene)/polystyrene sulfonate (PEDOT/PSS) polymer to fabricate a low pass filter that was operational below the MHz range [9,10]. In all cases the functional inks should exhibit low toxicity, good printability, and high mechanical/chemical stability. Furthermore, the ink synthesis methods should be simple and required equipment and raw materials must be financially feasible. Finally, the resultant printed thin film on a mechanically flexible substrate must exhibit acceptable electrical performance and excellent mechanical flexibility. 

Based on these requirements, liquid phase exfoliation of G is a practical approach for synthesis functional inks. Pristine G is highly hydrophobic and has been shown to be successfully suspended in a handful of organic solvents. *N*-Methyl-2-pyrrolidone (NMP) [11] and dimethylformamide (DMF) [12] are examples of commonly used organic solvents to suspend graphene nanoparticles. These organic solvents are toxic to human health [13], may affect fertility [14], and some of them are suspected to have carcinogenic properties [15]. Several attempts have been made to utilize GO, a heavily oxidized, hydrophilic, and electrically insulating derivative of G, to fabricate thin film circuits. GO films can be heat treated to remove oxygen containing moieties to form reduced GO (rGO) and restore conductivity [16,17]. Unfortunately, oxidation process permanently damages graphene lattice structure and fully reverting rGO to G is not possible at temperatures sustainable on flexible substrates.

In this research, a novel aqueous *carboxymethyl cellulose* (CMC) functionalized G ink has been developed to fabricate thin film circuits with inkjet printing technology. CMC is a non-toxic cellulose derivative that enables the naturally hydrophobic graphene flakes to be suspended in water solutions. CMC is also known to decompose efficiently under thermal treatment [18] which can facilitate its partial removal from deposited films and improve the electrical properties of thin films. Preliminary experiments have shown that the proposed G-CMC ink could be used to fabricate interdigitated capacitors (IDCs) and resistors on mechanically flexible polyimide [19]. Although the fabrication of individual passive components—such as electrodes, inductors, resistors (*R*) and capacitors (*C*)—is an important step, a deeper understanding of the combined response behavior of several interconnected components is essential for designing more complex inkjet-printed devices. Furthermore, the electrical behavior of fabricated G-CMC devices under mechanical strain needs to be investigated. In this study, the low pass electrical filters are designed according the principles outlined in Section 2. The ink synthesis and inkjet fabrication process are briefly summarized in Section 3. The electrically conductive and printable G-CMC ink is prepared in-house using non-toxic chemicals and scalable processes. In Section 4, the performance of these printed passive filters is compared to their conventional counterparts. Changes in the frequency response of the G-CMC printed devices are further analyzed under mechanical strain. Finally, concluding comments are made in Section 5.

## 2. Passive Low-Pass Electrical Filters

Electronic filters are designed using either passive components (e.g., resistors, capacitors, inductors) or active components (e.g., transistors, FETs and Op-amps). One of the most basic, and yet important, filters for communication and control circuits is the low-pass filter (LPF) that attenuates high frequency signals usually associated with carrier or noise. The cut-off frequency (*f_c_*) depends upon the components used in the design. For example, in low frequency applications (*f* < 100 kHz), passive filters are often constructed using simple resistor-capacitor (*RC*) circuits while for higher frequency low-pass filters (*f* > 100 kHz) these circuits are usually constructed using passive resistors, capacitors and inductors (*RLC*). 

**Figure 1:**
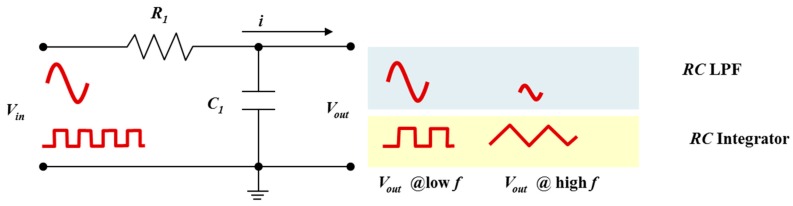
Passive first-order resistor-capacitor (*RC*) low-pass filter (RC-FO) for attenuating higher frequencies (*f* > *f_c_*) or performing wave shaping (i.e., *RC* integrator circuit).

The simplest LPF for low frequency applications is the first-order passive *RC* circuit shown in Figure 1. The resistance is independent to frequency variations in the a.c. signal whilst the capacitance changes with frequency. Since the circuit has only one reactive component, the capacitor, the circuit is called a one-pole or first-order filter. The capacitor requires a specified amount of time to charge and discharge through the resistor. At low signal frequencies there is sufficient time for the capacitor charge up to the input voltage (*V_in_*). However, at high frequencies the capacitor will not have enough time to fully charge before the input a.c. signal switches direction. To maintain the capacitance (*C*) value in the circuit, the capacitor will oppose these small fluctuations in current flow. The opposition to the current flow in the circuit is called impedance. In other words, the capacitive reactance (*X_C_*) is inversely proportional to the frequency (*f*) applied to the circuit and can be described as

(1) $$X_{C}=1/\left(2\pi fC\right)$$

Since the first-order *RC* LPF (RC-FO) is constructed from one resistor (*R*) and one capacitor (*C*), the overall impedance (*Z*) impedance can be calculated as

(2) $$Z=\sqrt{R^{2}+X_{C}^{2}}$$

where impedance, resistance, and capacitive reactance are all given in ohms (Ω). Note that *R* is stable and fixed whereas the *X_C_* varies with respect to the input voltage signal.

The voltage drop across the capacitor (*V_out_*) is dependent on capacitive reactance and circuit impedance. The *RC* potential voltage divider equation can be given as

(3) $$\frac{V_{out}}{V_{in}}=\frac{X_{C}}{\sqrt{R^{2}+X_{C}^{2}}}=\frac{X_{C}}{Z}$$

where *V_in_* is the input voltage signal. By using Equation (3) it is possible to calculate *V_out_* at any applied frequency (*f*). 

The magnitude of the gain of the first-order *RC* LPF in decibels is given as |*H*(*f*)|*_dB_* and can be calculated as

(4) $${\left|H\left(f\right)\right|}_{dB}=20\log\frac{V_{out}}{V_{in}}$$

The frequency at which the output of the low pass filter is reduced by 3 dB is called the cut-off frequency (*f_c_*). The *f_c_* for the electrical filter depends on the values of resistor and capacitor and can be written as

(5) $$f_{c}=\frac{1}{2\pi RC}$$

where *C* is given in Farads, *R* in Ohms and *f_c_* in Hz. At *f_c_* the output signal (*V_out_*) is 70.7% of the input signal (*V_in_*), and gradually decreases to zero as frequencies increase. This cut off point will occur when the resistance and the capacitive reactance are equal (*R* = *X_C_*) and, at this point, the input signal is attenuated by −3 dB/decade. The gain will further decrease along with the output voltage after the cut off frequency where the slope reaches a roll-off point that occurs at Δ*L* = −20 dB/decade. 

The time constant (*τ*) of a series *RC* circuit is defined as the time taken by the capacitor to charge up to 63.2% of the final steady state value and given by

(6) $$\tau =1/\left(2\pi f_{c}\right)=RC$$

Note that the time constant is also defined as the time taken by the capacitor to discharge to 36.8% of steady state value.

In addition to attenuating high frequency sinusoidal signals, the basic *RC* LPF can act as a wave shaping circuit for a square wave input. This type of LPF circuit, sometimes called a *RC* integrator circuit, can convert the square wave to a triangular waveform. At low frequencies the output signal will emulate the square wave input. However, as the frequency increases the output waveform takes on a triangular shape because of the capacitor’s inability to instantly charge and discharge. The amplitude of the output signal will also decrease. If the *τ* is long compared to the time period of the input waveform (*V_in_*), then the resultant output waveform (*V_out_*) will be triangular in shape and the higher the input frequency the lower will be the output amplitude compared to input.

**Figure 2:**
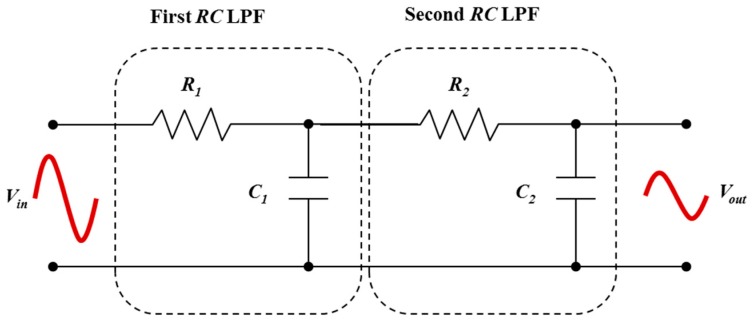
Illustration of cascading two first-order *RC* low pass filters (RC-FO1 and RC-FO2) to obtain a second-order *RC* low pass filter (RC-SO).

Sometimes a single stage LPF may not enough to remove all unwanted frequencies, then second-order filters are used (Figure 2). The second-order *RC* low pass filters (RC-SO) are constructed by cascading two first-order *RC* LPFs. In contrast to a first-order *RC* LPF, this type of filter produces a slope of −40 dB/decade. It is possible to create even higher-order filters by connecting more first-order *RC* LPFs resulting in a *RC* filter circuit known as an *n*^th^-order LPF with a roll-off slope of

(7) $$\Delta L_{Total}=n\Delta L=n\left(-20\;\mathrm{dB}/\mathrm{decade}\right)$$

Unfortunately, these higher-order *RC* LPFs can introduce problems because the gain and accuracy of the final filter design declines with increasing order. When identical *RC* LFPs are cascaded in series, the output gain at the required cut-off frequency (*f_c_*) is attenuated and the roll-off slope Δ*L_Total_* increases in relation to the number of stages (*n*). For example, the cut-off frequency of a second-order *RC* low-pass filter (i.e., RC-SO) is given as

(8) $$f_{c}=\frac{1}{2\pi \sqrt{R_{1}C_{1}R_{2}C_{2}}}$$

Note that cascading passive *RC* LPFs to produce larger-order filters is difficult to accurately create because the dynamic impedance of each constituent filter will affect the neighboring filters in the network. However, to reduce the impact of loading it is possible to adjust the impedance of each successive stage by a factor of 10; that is *R*_2_ = 10*R*_1_ and *C*_2_ = *C*_1_/10. 

In terms of printable electronics, the resistors are often given as conductive traces where the resistivity varies with geometrical dimensions such as length, width and film thickness. Often, increase in electrical resistivity is achieved by increasing the length of the trace. It is important to realize, however, that the droplet deposition printing methods do not create uniform thin films similar to those achieved using photolithography or chemical vapor deposition (CVD) methods. Consequently, highly precise resistance values are nearly impossible to obtain with inkjet printing.

**Figure 3:**
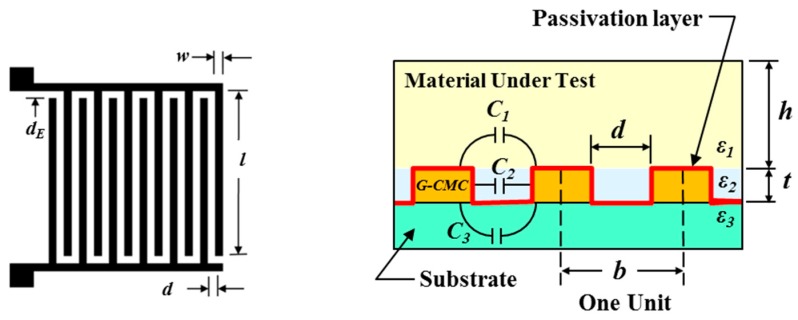
Basic layout design and cross-sectional view of a planar graphene-carboxymethyl cellulose (G-CMC) interdigitated capacitor (IDC) that responds to dielectric changes in the material under test (MUT).

There are different approaches to creating capacitors, but the most common printable design is the two-dimensional interdigitated capacitor (IDC). The IDC has a planar structure where the consecutive fingers are connected to positive and negative electrodes (Figure 3). The capacitance is dependent upon the dielectric properties of the electrode surface and can change when the device is exposed to an external influence (e.g., material under test). The IDC electrodes can be printed directly on the substrate material and coated with a very thin passivation layer to prevent an electrical short when encountering the material under test (MUT). Changes in the MUT will alter the measured capacitance. For signal processing applications it is important to electrically isolate the capacitor to minimize the impact of environmental changes (i.e., *C_MUT_*→0).

From a design perspective, the *total capacitance* (*C_IDC_*) of the IDC can be estimated by

(9) $$C_{IDC}=l\left(n-1\right)\left(C_{MUT}+C_{Passivation}+C_{Substrate}\right)$$

where *l* is the length of the finger electrode, *n* is the number of digits, and *C_MUT_*, *C_Passivation_*, and *C_Substrate_* are the partial capacitances due to the MUT, passivation layer and substrate, respectively.

The partial capacitance values are based on material permittivity and can be calculated as

(10) $$C_{MUT}+C_{Substrate}=\varepsilon_{0}\left(\frac{\varepsilon_{MUT}+\varepsilon_{Substrate}}{2}\right)\frac{K\left[\sqrt{1-{\left(\frac{d}{b}\right)}^{2}}\right]}{K\left[\frac{d}{b}\right]}$$

(11) $$C_{Passivation}=\varepsilon_{0}\varepsilon_{Passivation}\left(\frac{t}{d}\right)$$

where *d* is the gap between adjacent electrodes, *b* is the distance between electrode centers, *t* is the thickness of the printed G-CMC electrode, *ε*_0_ is the permittivity of free space, *ε_MUT_* is the relative permittivity of the MUT, *ε_Passivation_* is the relative permittivity of the passivation layer, and *ε_Substrate_* is the relative permittivity of the polydimethylsiloxane (PDMS) substrate. The function *K*[*x*] is determined by using the complete elliptic integral of the first kind because it provides a good model for magnetic fields.

The total capacitance of the IDC will increase with smaller gaps (*d* and *d_E_*) but the minimum gap size is dictated by the smallest reproducible feature size. For inkjet printed circuits the minimum feature size is dependent, in large part, upon the nozzle diameter and resolution of the XY positioning system. The thickness of the conductor material (*t*) and its electrical resistivity (*ρ*) will also affect circuit performance. However, controlling the thickness of an inkjet-printed film is a challenge because of the inconsistent droplet deposition process. Finally, increasing the length of the fingers (*l*) will increase the overall capacitance but will significantly enlarge the physical dimensions of the printed IDC.

## 3. Materials and Methods 

### 3.1. Ink Synthesis

To synthesize G-CMC ink, 2 mg/mL 250 kDa CMC was dissolved in DI water under magnetic stirring. Into this solution, G nanoparticles (Graphene Supermarket-A12) were mixed at a concentration of 2 mg/mL. After a brief and gentle shake, the mixture was sonicated for 8 h in a 110 W output bath ultra-sonicator. Resulting G-CMC suspension was centrifuged at 2000 *g* for 15 min to precipitate large and unstable particles. This step was repeated for a second time. After sonication and centrifuge steps, some amount of free CMC persists in the solution. This unbound CMC can negatively affect film properties. To remove free CMC, the solution was centrifuged at 20,000 *g* for 30 min and supernatant was disposed. Sediment left at the bottom of centrifuge tube was re-suspended in DI water. High speed centrifuge step was repeated one more time to ensure complete removal of free CMC. Finally, the second step sediment was re-suspended in DI water at 4 times the initial concentration.

**Figure 4:**
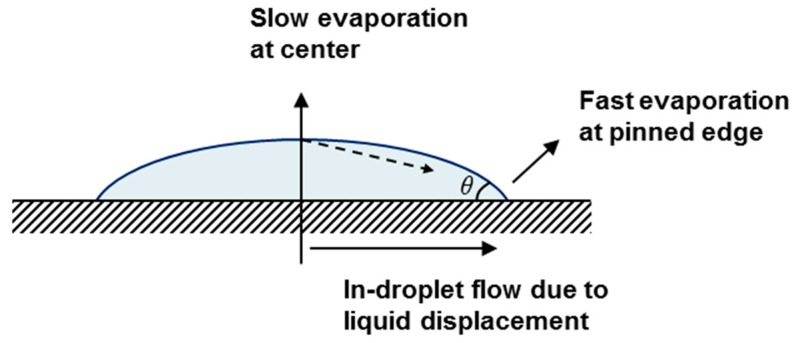
Flow towards droplet edges can result in the coffee ring effect (CRE). Dotted arrows show the direction of initial flow. Due to decreasing *θ* angle during evaporation, liquid displacement from center to edge occurs. On the other hand, if initial contact angle *θ* is sufficiently small and there is no surface tension gradient between center and edge, droplet can evaporate with minimal in-droplet flow towards edges.

There are several frequently encountered problems with inkjet deposition of materials. The most common problems are poor wetting and coffee ring effect (CRE). Poor wetting is usually encountered with water based inks. Water has low viscosity (~1 cP) and very high surface tension (~70 mN/m). In comparison, most flexible plastic substrates have relatively lower surface energy values that can range from 40 (Polyimide) to 18 (PTFE) mN/m. As a result, poor wetting and quick recession of contact line can occur when printing with plain water. It is also necessary to prevent formation of edge heavy deposition of particles which is commonly referred to as CRE (Figure 4). CRE happens when a droplet has pinned contact lines and is evaporating [20]. Because droplet edges evaporate faster due to larger surface area and contact line cannot recede, liquid from droplet center has no choice but to flow towards the edges to maintain reduction in contact angle *θ*. This flow carries colloid particles and results in distorted film deposition, resembling coffee stains on a surface. Conversely, if contact lines can recede, contact angle *θ* would not change and flow towards the edges would not be necessary. Both poor wetting and CRE can be addressed by choosing the correct solvent. Hence, a binary solvent system was proposed previously [19] where low rate of change in surface tension of binary system during evaporation can keep edge directed Marangoni flow under control. N-Propanol/water binary system has a relatively stable surface tension profile between 20% m/m to 100% m/m N-Propanol. As a result, surface tension gradient between droplet center and edge differs only slightly during evaporation. Moreover, smaller *θ* angle means less difference between evaporation rate of droplet center and edge. Hence, it was observed that surface tension gradient was not large enough to exacerbate CRE and films with good morphology could be achieved when 28% m/m N-Propanol was present in G ink. Based on this, all the devices presented in this study were inkjet printed with G-CMC ink containing 28% m/m N-Propanol.

### 3.2. Inkjet Printing of Passive Electrical Filters

For this investigative study, all graphene-based passive *RC* filters were fabricated through using a custom-made inkjet printer. The printer features an XY positioning system capable of 2.5 µm step size and 4800 mm/min feedrate. G-CMC ink was deposited by HP6602A print head. This print head is capable of jetting 160 pl of ink per droplet. Droplets were spaced 25 µm and 180 µm in vertical and horizontal directions, respectively. All circuitry was printed 10 times. Based on previous data, this would correspond to approximately 250 to 300 nm film thickness. Substrates (HN polyimide) were cleaned by ethanol/water wash and air dried. Cleaned substrates were corona treated briefly and submerged in a polyethyleneimine solution (0.75 g/100 mL PEI + 2.95 g/100 mL NaCl) for 20 min. Substrates were washed with DI water, air dried, and submerged into a solution of Polystyrene 4-Sulfonate (0.9 g/100 mL PS + 2.95 g/100 mL NaCl) for 20 min. Finally, substrates were washed with DI water, air dried, and submerged in PEI solution one last time. Resulting polyelectrolyte coating on polyimide substrates facilitated homogeneous self-assembly of deposited particles.

Printed circuits were heat treated in an Argon environment at 320 °C for 30 min to partially decompose the CMC. Note that decomposition of CMC increases conductivity roughly 2 times. Once heat treatment was finished, circuits were covered with a second layer of polyimide with silicone adhesive on the side facing the *RC* components. Exposed contact pads were covered with small amount of silver paste to allow stable connection to Agilent arbitrary waveform generator and Agilent DSO oscilloscope.

**Figure 5:**
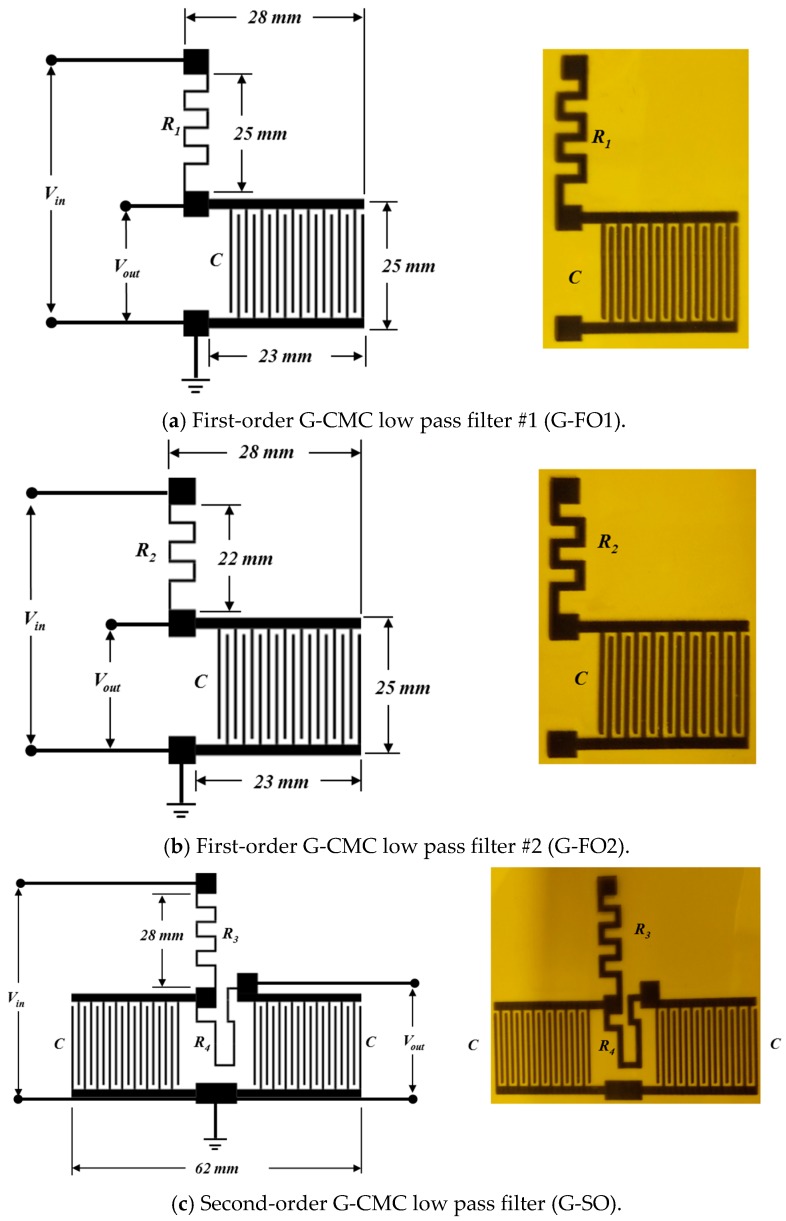
First-order G-CMC low pass filters (**a**) G-FO1 and (**b**) G-FO2, and second-order G-CMC low pass filter (**c**) G-SO circuits and the corresponding inkjet-printed devices based on these designs.

Several passive filters were inkjet-printed for analysis: first-order G-CMC low pass filter #1 (G-FO1), first-order G-CMC low pass filter #2 (G-FO2), and second-order G-CMC low pass filter (G-SO). The two first-order LPFs were variant designs. For G-FO2 and G-SO, filter circuits were fabricated with a different *R* values to demonstrate the degree of control on *f_c_*. The *R*, *C*, *τ* and *f_c_* values for each inkjet-printed G-CMC filter are summarized in Table 1. Schematics of these all filter circuits are depicted in Figure 5. Each filter shares the same basic IDC capacitor design with 18 electrode fingers where *w* = 0.5 mm, *d* = 0.5 mm and *l* = 20 mm. The entire IDC covers an area of 30 × 28 mm^2^. The resistor *R* for variant designs was adjusted by simply increasing length and width of the resistive trace. The printed trace for the *R*_1_ resistor was 48 mm × 1.8 mm while *R*_2_ resistor was 43 mm × 1.8 mm. *R*_3_ and *R*_4_ resistors were 55 mm × 1.3 mm and 45 mm × 1 mm, respectively. Slight dimensional variations were observed in the actual printed films and these variations resulted in a ~2% difference from the desired resistance values. Note that from a design perspective, the resistance of the printed trace will increase with length or reduction in film thickness. For comparative purposes, two additional filter circuits were constructed on a breadboard using conventional *R* and *C* components. *R* and *C* component values of these circuits were chosen to closely match the *R* and *C* components of G-CMC based devices. 

## 4. Results

**Figure 6:**
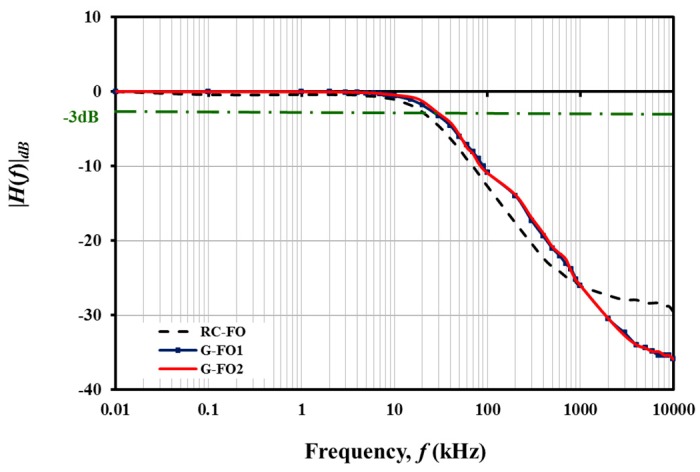
First-order low pass filter performance of RC-FO, G-FO1 and G-FO2. Note that the *f_c_* was same for both G-CMC circuits and roll off rate was lower than the conventional *RC* low pass filter.

The frequency response of the passive filter circuits was analyzed by supplying a sinusoidal signal with 1 V_pp_ amplitude. The output voltage was measured with the oscilloscope and mean *V_out_* was recorded for each data point (10 Hz to 1 MHz). G-CMC based low pass filters exhibited diminishing *V_out_* at their designated cut-off frequency (*f_c_*). G-FO1 had a calculated *f_c_* of ~30.5 kHz while the variant G-FO2 was fabricated with a different *R* value to observe the sensitivity of the device to changing component parameters. The *f_c_* of G-FO2 was calculated to be 27.8 kHz. As expected, the measured *f_c_* of the G-FO2 filter had shifted due to the larger *R*. Both G-FO1 and G-FO2 performed satisfactorily as low pass electrical filters (Figure 6). A conventional *RC* passive filter (RC-FO) was also constructed using the same *R* and *C* values for G-FO1. The frequency response of RC-FO and G-FO1 did not differ significantly with both filters exhibiting diminishing *V_out_* near their designated *f_c_*. The roll-off rate was essentially identical for both G-FO1 and G-FO2, and slightly less than RC-FO (Table 2). However, modest irregularities in the gain were observed between 100 kHz and 1 MHz for both G-FO1 and G-FO2. The nonlinearities arise, in part, from the dimensional variations in the geometric parameters of the printed IDC capacitor which determine the natural resonance frequency of the device. Small variations in the fabricated IDC capacitor due to the droplet deposition method (i.e., inkjet printing process) will produce nonlinearities in the frequency response. It is likely that the increase in capacitance near the resonant frequency of the printed IDC capacitor may have contributed to the slight observed shift in gain between the conventional *RC* (RC-FO) and printed (G-FO1 and G-FO2) filter circuits.

**Figure 7:**
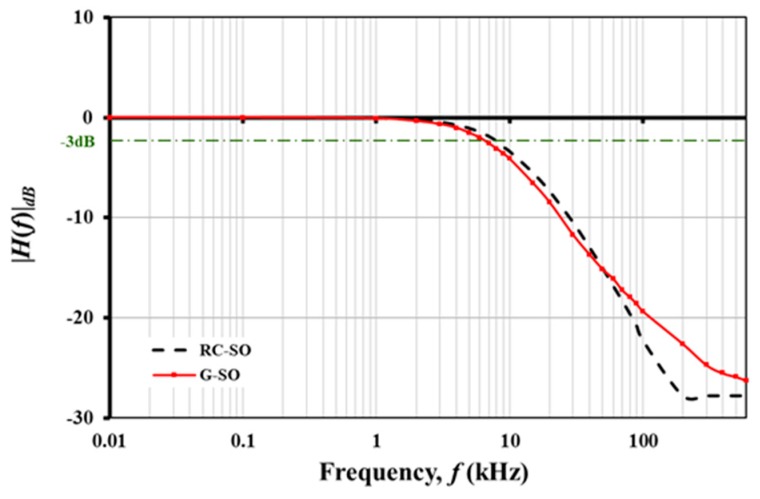
Comparison of second-order G-CMC low pass filter performance with equivalent conventional *RC* circuit. Note that the second order filter roll-off rate was roughly twice of first order circuit. As expected, *f_c_* value did not differ between first and second-order low pass filters. The output voltage signal was detectable beyond 600 kHz.

A second-order passive filter (G-SO) was also fabricated and characterized separately (Figure 7). The calculated *f_c_* for the G-SO was around 8 kHz. A small decrease was observed for G-SO *V_out_*. Similar irregularity was also observed for second-order filter constructed from conventional *RC* components (RC-SO) as well, albeit at a smaller magnitude. The roll-off rates of the G-SO and RC-SO filters were 15.2 and 18.8 dB/decade, respectively. The *f_c_* of G-SO showed a slight shift (approximately 1 kHz) compared to the RC-SO despite similar component values.

**Figure 8:**
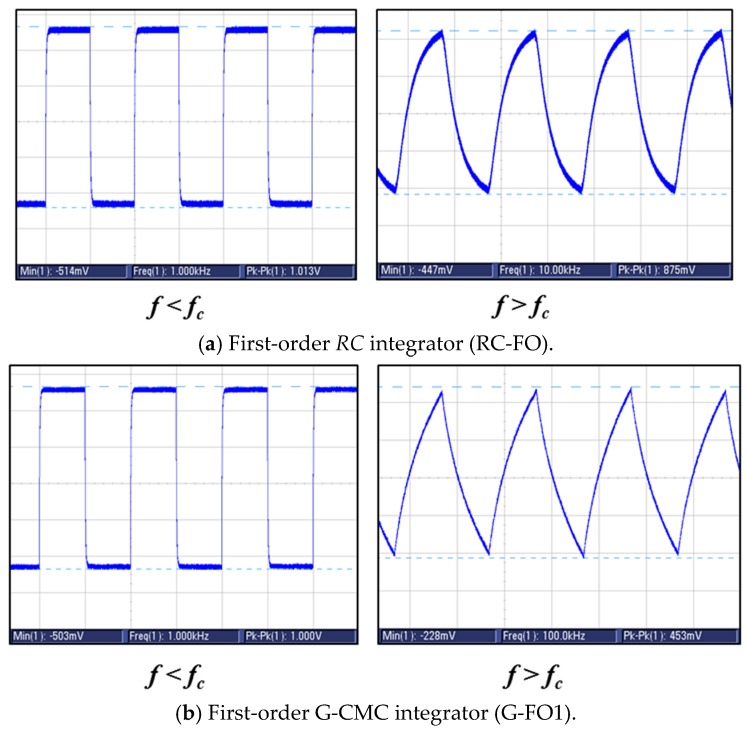

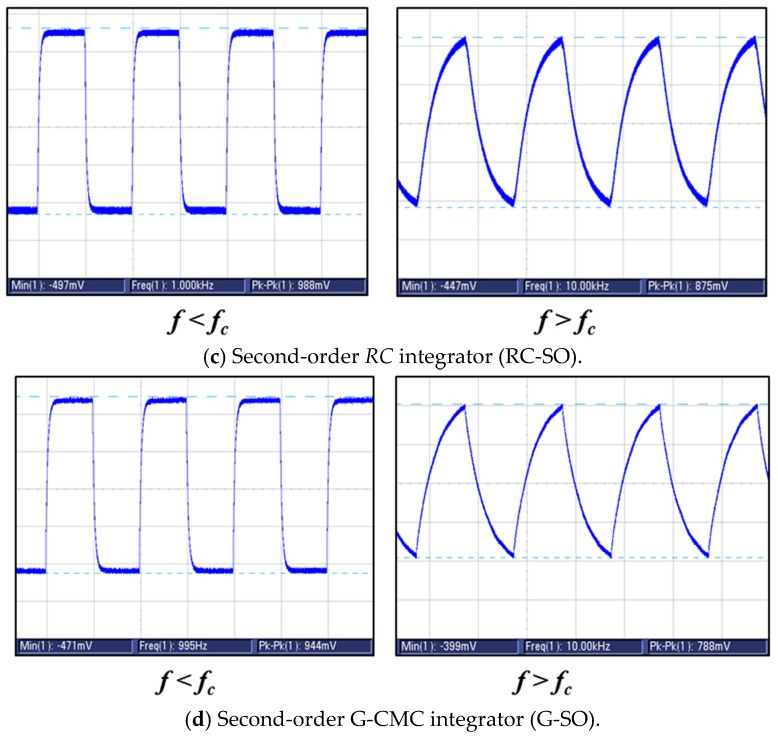
Comparison of transient response for (**a**) first-order *RC* integrator; (**b**) first-order G-CMC integrator; (**c**) second-order *RC* integrator, and (**d**) second-order G-CMC integrator. As expected, output signal was a square wave (left-side) below *f_c_*. Beyond *f_c_*, the signal was converted to a triangular wave (right-side).

The transient response of both G-CMC and conventional passive electrical filters was also investigated using a square wave with 1V amplitude. Transient response was observed for both low (1 kHz) and high frequency regimes. For high frequency regime, which is defined here as frequencies at and beyond *f_c_*, square wave inputs were effectively converted to triangular signals for both graphene and *RC* conventional filters. Hence, all filters performed as expected from *RC* integrator circuits. Change in waveform below and above *f_c_* can be observed in Figure 8.

**Figure 9:**
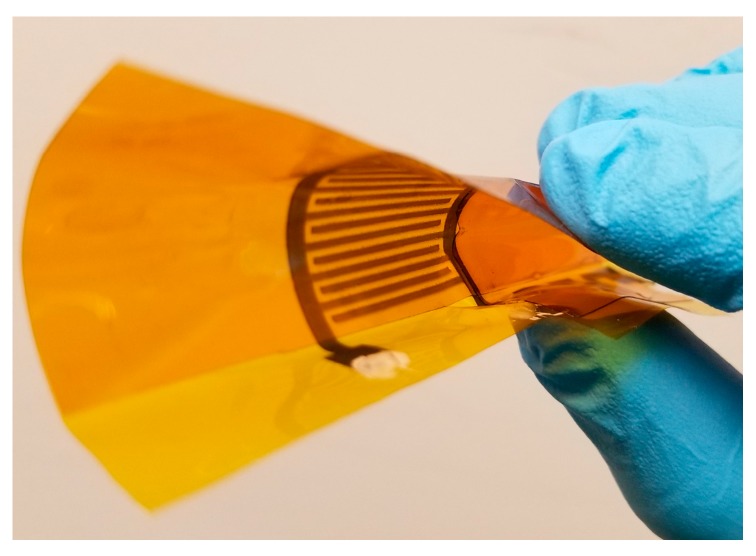
G-CMC filter devices were rolled into a cylinder during mechanical stress tests. There were no measurable differences in frequency response behavior between rolled and unrolled states. As expected, the *R* and *C* component values of the individual filters were also unaffected from rolling.

Finally, the inkjet-printed low pass filters were tested under mechanical stress. Inkjet-printed G-CMC films have already been shown to exhibit excellent flexibility under mechanical stress [19,21]. Shape conformable and wearable electronics should not only perform under mechanical stress but also behave in predictable fashion. In the case of passive filters, a large variation in capacitance and/or resistance of the circuit would cause a large shift in *f_c_* during high strain. This could potentially cause device failures. To assess the *f_c_* error margins due to bending stress, each device was analyzed while being rolled into a cylinder (Figure 9). The frequency response characteristics for each G-CMC printed filter (G-FO1, G-FO2, and G-SO) showed no observable changes while under strain. 

**Figure 10:**
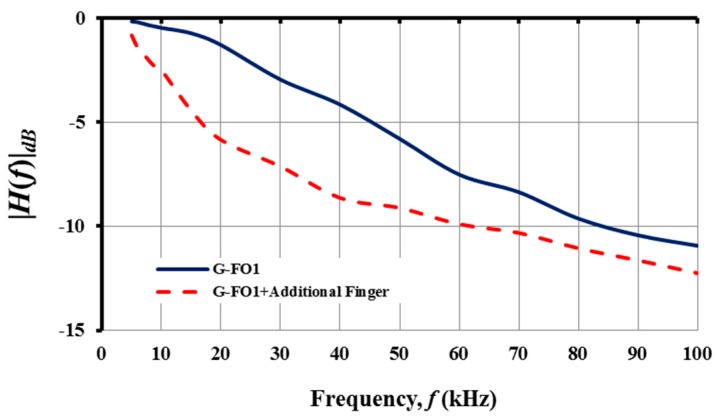
Change in the frequency response of the G-FO1 filter when capacitance is increased by introducing a foreign object to the IDC. Note that *f_c_* of G-FO1 is shifted from 30.5 kHz to 5 kHz when a finger is placed on the IDC component.

In contrast, it was possible to manipulate the frequency response of the filters by placing a foreign object, such as the finger, on the surface of the IDC capacitor. The shift in the frequency response behavior, as shown in Figure 10, is expected because the capacitance of the IDC is based on the dielectric properties of the printed film, substrate and surrounding media. For a finger placed on the IDC of the G-FO1 filter circuit, the cut-off frequency *f_c_* decreased by ~5 kHz. In addition to increasing the capacitance of the IDC component, the foreign object (i.e., human finger) may have introduced stray capacitance and inductance that impacted the filter circuit and decreased the observed roll-off rate. The variations in dielectric properties of the surrounding media clearly change the response characteristics of passive filters constructed from printed IDCs. Future research activities will need to focus on the impact of dielectric sensing on the filter characteristics. 

## 5. Discussion

In this study, several passive resistor-capacitor (RC) electrical filters were printed on mechanically flexible polyimide substrates using a customized inkjet printer and synthesized non-toxic aqueous graphene-carboxymethyl cellulose (G-CMC) ink. The fabrication and post-processing of electronic devices were achieved using simple tools; graphics software for device design, XY plotter style printer with HP6602A print head for ink droplet deposition, and a low temperature treatment to decompose the CMC in the printed films. The various G-CMC printed filters exhibited frequency response and transient behavior similar to conventional discrete component *RC* filter circuits. Minor variations arose because the printed circuit designs incorporated two-dimensional interdigitated capacitors (IDCs) instead of traditional parallel plate capacitors. Adjusting the design parameters enabled filters with different cut-off frequencies and roll-off rates to be fabricated. However, the quality of the printed films created for the circuits were influenced by the limitations of the inkjet printing process. Inkjet printing is a controlled droplet deposition method that produces a very different type of film than chemical vapor deposition (CVD) or photolithography. The print head nozzle size, resolution of the XY positioning system and droplet speed all play a role in the physical structure and dimensional accuracy of the resultant conductive film. Droplet deposition methods also require multiple passes over the same feature to ensure a contiguous film for unimpeded flow of electric charge. These factors will impact the observed electrical behavior of any inkjet printed circuit or sensor. In this regard, the conductive ink and printing methodology described in this paper is best suited for flexible electronics that do not require high precision. A further advantage is that the water based ink is synthesized from non-toxic, and likely biocompatible, materials. This presents new opportunities for novel wearable technologies or dissolvable electronics.

The proposed ink synthesis and droplet deposition methods are also transferable to other types of engineered materials, such as doped graphene (DG) and graphene/metal composite inks. It has been shown in past studies that the electrical conductivity of G-CMC films can be significantly increased through gold chloride (AuCl_3_) doping [22] or directly printing G-CMC/silver nanoprism (AgNP) composite inks [23]. In addition, N-type DG has been shown to possess favorable electrical properties [24] and the combined use of G and GO, which is considered a P doped material, has been used to create a Schottky barrier [25]. In this context, it may be possible to fabricate highly efficient optically transparent electrodes or simple diodes by printing various combinations of DG, G-AgNP, and G-CMC inks.

One of the greatest challenges in fabricating sophisticated electronic circuitry is overcoming the limitations of the inkjet printing process. The minimum line width, or feature size, that an advanced material printer can achieve is ~25 µm. Large character print heads, such as the HP6602A, tend to deposit larger ink droplets and therefore produce bigger features. During this study, it was observed that the minimum line width could be as large as 500 µm, depending on substrate properties. Fidelity to design dimensions was also problematic due to the large droplet size. However, using alternative commercial printers with smaller droplet volumes (33 pl for HP54 print head) could allow for printing thinner and more robust lines. Nevertheless, the extremely small features achieved using lithography techniques are currently impossible with direct inkjet printing. To address these dimensional limitations, it is possible to combine inkjet printing and micromachining (e.g., laser or micromilling). Previous research has shown that it is possible to micromachine 8 µm gaps on rGO films using a 120 femtosecond laser [21]. The micromachining operation was performed on polyimide substrates and no damage to polyimide was experimentally observed. Theoretically, it should also be possible to micromachine highly robust submicron features G-CMC films with a well optimized pulsed laser system. This would increase robustness of fabricated devices and substantially reduce error margin that stems from otherwise poorly controlled thin film geometry. Furthermore, by laser annealing, the heat treatment step can be eliminated, which would make it possible print on other types of plastics. Based on these preliminary observations, future work will focus on laser heating and micromachining of very fine features on G-CMC inkjet-printed films.

## Figures and Tables

**Table 1:** Resistance (*R*), capacitance (*C*), time constant (*τ*) and cut-off frequency (*f_c_*) for printed G-CMC passive filters.

Component	G-FO1	G-FO2	G-SO
*R* (kΩ)	290	317.5	520
*C* (pF)	18	18	18
*τ* (μs)	5.22	5.72	9.36
*f_c_* (kHz)	30.5	27.8	8

**Table 2:** Roll-off rate comparison of graphene-CMC passive filters.

Roll-Off Rate (dB/Decade)	G-FO	G-SO
Off-the-Shelf *RC* Circuit	15.8	18.8
G-CMC Printed *RC* Circuit	14.4	15.2
